# Prevalence and antimicrobial susceptibility pattern of methicillin resistant *Staphylococcus aureus *isolates from Trinidad & Tobago

**DOI:** 10.1186/1476-0711-5-16

**Published:** 2006-07-03

**Authors:** Patrick Eberechi Akpaka, Shivnarine Kissoon, William Henry Swanston, Michele Monteil

**Affiliations:** 1 Department of Paraclinical Sciences, Faculty of Medical Sciences, The University of the West Indies, St. Augustine, Trinidad & Tobago

## Abstract

**Background:**

Methicillin-resistant *Staphylococcus aureus *(MRSA) has become increasingly prevalent worldwide since it was first reported in a British hospital. The prevalence however, varies markedly in hospitals in the same country, and from one country to another. We therefore sought to document comprehensively the prevalence and antimicrobial susceptibility pattern of MRSA isolates in Trinidad and Tobago.

**Methods:**

All *Staphylococcus aureus *isolates encountered in routine clinical specimens received at major hospitals in the country between 2000 and 2001 were identified morphologically and biochemically by standard laboratory procedures including latex agglutination test (Staphaurex Plus; Murex Diagnostics Ltd; Dartford, England); tube coagulase test with rabbit plasma (Becton, Dickinson & Co; Sparks, MD, USA), and DNase test using DNase agar (Oxoid Ltd; Basingstoke, Hampshire, England). MRSA screening was performed using Mueller-Hinton agar containing 6 μg oxacillin and 4% NaCl, latex agglutination test (Denka Seiken Co. Ltd, Tokyo, Japan) and E-test system (AB Biodisk, Solna, Sweden). Susceptibility to antimicrobial agents was determined by the modified Kirby Bauer disc diffusion method while methicillin MICs were determined with E-test system.

**Results:**

Of 1,912 *S. aureus *isolates received, 12.8% were methicillin (oxacillin) resistant. Majority of the isolates were recovered from wound swabs (86.9%) and the least in urine (0.4%) specimens. Highest number of isolates was encountered in the surgical (62.3%) and the least from obstetrics and gynaecology (1.6%) facilities respectively. Large proportions of methicillin sensitive isolates are >85% sensitive to commonly used and available antimicrobials in the country. All MRSA isolates were resistant to ceftriaxone, erythromycin, gentamicin and penicillin but were 100% sensitive to vancomycin, rifampin and chloramphenicol.

**Conclusion:**

There is a progressive increase in MRSA prevalence in the country but the present rate is still low in comparison to values in some other countries. Vancomycin is still the drug of choice for treating multidrug resistant MRSA infections. Further use of molecular studies to monitor the epidemiology of MRSA in these hospitals in the country is highly recommended too.

## Background

Methicillin-resistant *Staphylococcus aureus *(MRSA) has become increasingly prevalent worldwide since it was first reported among hospitalized patients in a British hospital [1,2]. The prevalence of MRSA as a major cause of morbidity, increased cost of healthcare delivery services and mortality in hospitals has increased during the last decade [3-5]. The prevalence of MRSA differs widely among different countries including from one hospital to another in the same country [6]. In the USA for instance, the prevalence of MRSA in some areas initially used to be associated with tertiary care referral hospitals affiliated perhaps with teaching hospitals [7,8], but it is now present in hospitals of all sizes [9].

In many hospitals and areas worldwide, the prevalence of MRSA poses a serious therapeutic problem. There are limited choices of antimicrobial agents to treat many serious life-threatening infections caused by MRSA leading to prolonged stay of such patients in the hospital and increased cost of care. In Trinidad and Tobago, several independent studies of MRSA prevalence though limited in many aspects have been carried out in one hospital or the other in the country. Those previous studies reported the prevalence of MRSA from a cross section of one hospital or another in the country and this may not have reflected the true picture of the general prevalence rate of MRSA existing in Trinidad & Tobago. We therefore sought to determine in a more comprehensive manner the prevalence and antimicrobial susceptibility pattern of MRSA in three major regional hospitals in the country.

## Methods

### Source of bacterial isolates and identification

*Staphylococcus aureus *isolates from routine clinical specimens submitted at the microbiology laboratories of three major regional hospitals (from the south, San Fernando General Hospital SFGH; from northwest, Port of Spain General Hospital POSGH and from north central, Eric Williams Medical Sciences Complex, EWMSC) in the country from January 2000 to December 2001 were included in this study. All isolates were identified morphologically and biochemically by standard laboratory procedures [10]. No duplicate isolates from a single patient were included. The isolates were identified using latex agglutination test (Staphaurex Plus; Murex Diagnostics Ltd; Dartford, England), tube coagulase test with rabbit plasma (Becton, Dickinson & Co; Sparks, MD, USA), and DNAse test with DNAse agar plate (DNase, Oxoid Ltd; Basingstoke, Hampshire, England).

### Methicillin resistance detection

#### (1) Oxacillin disk diffusion tests

The entire surface of Mueller-Hinton agar (MHA) plate containing 6 μg oxacillin and 4% NaCl was covered with the required inoculum, and the plate was air dried before a 1 μg disks were laid on the surface. The plates were incubated in ambient air atmosphere for 18 – 24 hours at 35°C. Evidence of growth suggested that the isolates were oxacillin resistant.

#### (2) MRSA-Screen latex agglutination test

The MRSA-Screen latex agglutination test (Denka Seiken Co. Ltd, Tokyo, Japan) was performed according to the manufacturer instructions. For each strain, a 5-μl loopful of *S. aureus *colonies was obtained from fresh subculture and was suspended in 1.5 ml micro centrifuge tube containing 200 μl of extraction reagent # 1 (0.1 M NaOH). The suspension was boiled for 3 minutes and then one drop (50 μl) of extraction reagent #2 (0.5 N KH_2_PO_4_) was added and mixed well. The mixture was centrifuged at 1500 × g for 5 minutes at room temperature. A 50 μl aliquot of the supernatant was added to each of the two circles on a disposable test card and mixed with one drop (25 μl) of the anti-PBP 2a monoclonal antibody sensitized latex and one drop (25 μl) of the negative control latex, respectively. The contents on the slide were then mixed for 3 minutes on a shaker and agglutination was observed visually and was scored as positive, negative or weakly positive

#### (3). E – test method

Methicillin (oxacillin) MICs were determined with the E-test strips (AB Biodisk, Solna, Sweden) using 0.5 McFarland inoculum according to the manufacturer's instructions. The oxacillin E-test strip was placed onto Mueller-Hinton agar plate supplemented with 2% NaCl, and the plate was incubated at 35°C for 24 h. Methicillin susceptibility and methicillin resistance were defined as oxacillin (OXA) E-test MICs of = 2 and = 4 μg/ml respectively. All results were validated using quality control strains: MRSA ATCC 43300, MSSA ATCC 25923 and *S*. *epidermis *ATCC 12228

### Antimicrobial susceptibility and MIC testing

Susceptibilities to common antibiotics were determined by modified Kirby-Bauer disk diffusion methods according to the Clinical Laboratory Standards Institute, formerly NCCLS guidelines [11]. The following antibiotics disks: ceftriaxone (30 μg), chloramphenicol (30 μg), ciprofloxacin (5 μg), erythromycin (15 μg), gentamicin (10 μg), meropenem (10 μg), oxacillin (1 μg), penicillin (10 u), tetracycline (30 μg), trimethoprim-sulphamethaoxazole (1.25/23.75 or 25 μg), rifampin (10 μg) and vancomycin (30 μg); all from Oxoid (Oxoid Ltd; Basingstoke, Hampshire, England) were tested. The MICs of the isolates were confirmed with the E-test methods as above.

Parameters such as increasing inoculum, prolonged incubation from 18 hours – 24 hours, growth at low temperature of 35°C and oxacillin screen test with NaCl were employed in certain cases to improve sensitivity or specificity for the tests methods. All tests gave satisfactory results with the reference strains.

## Results

A total of 1912 *Staphylococcus aureus *isolates were received from the three major hospitals. The performance of all three tests methods used to detect (screen) methicillin resistance in the *Staphylococcus aureus *isolates revealed very satisfactory results. Oxacillin disk diffusion and Latex agglutination methods compared excellently well with the E-test methods which provided a very rapid method for measuring the MICs for all the S. aureus isolates. All tests methods had 100% sensitivity and specificity for all isolates identified as MRSA or methicillin sensitive *S. aureus *(MSSA).

Results of specimen source and distribution of the MRSA isolates in the different facility of the three major hospitals in Trinidad & Tobago are depicted in Tables 1 &2. Half (50%) of the MRSA isolates were from the San Fernando General hospital. Majority of the isolates were recovered from wound swabs (86.9%) and the surgical facility (62.3%) of each hospital while the least were from urine (0.4%) and the obstetrics and gynaecology (1.6%) facility. There was no significant difference in the gender distribution of the *S. aureus isolates *in terms of their susceptibility to methicillin. Among the MSSA isolates male patients accounted for 49% (817/1668) while it was 51% (851/1668) for females; and for MRSA isolates it was 49.6% (121/244) for males and 50.4% (123/244) for females.

**Table 1:** Specimen source distribution of MRSA clinical isolates from three major hospitals in Trinidad & Tobago, 2000 – 2001

Hospital	N	Specimen source of MRSA isolate (%)					
		
		Blood	W/swabs	Sputum	Eye swab	Urine	others
POSGH	117(48)	2	105	3	2	1	4
EWMSC	6(2)	0	6	0	0	0	0
SFGH	121(50)	2	101	3	3	0	12

Total	244	4(1.6)	212(86.9)	6(2.5)	5(2.0)	1(0.4)	16(6.6)

N = total number of isolates; W/swabs = wound swabs; POSGH = Port of Spain General Hospital; EWMSC = Eric Williams Medical Science Complex; SFGH = San Fernando General Hospital; Others = tubes and fluid types specimens

**Table 2:** Facility distribution of MRSA isolates from three major regional hospitals in Trinidad & Tobago, 2000–2001.

Hospital	facility from which MRSA was isolated (%)					
	
	Surgery	medicine	paediatrics	ICU	O&G	outpatient
POSGH	65	34	3	8	1	6
EWMSC	3	0	2	0	0	1
SFGH	84	11	15	7	3	1

Total	152(62.3)	45(18.5)	20(8.2)	15(6.1)	4(1.6)	8(3.3)

ICU = Intensive Care Unit; Outpatient = includes isolates from patients coming from outpatient clinics, district health centers, private GPs and office; O & G = Obstetrics and Gynecology

The E-test oxacillin tests revealed that 87.2% (1668/1912) of the isolates had MICs of <2 mg/ml, 1.8% (34/1912) had values >64 mg/ml and 11% (210/1912) were >256 mg/ml; and oxacillin resistance (MICs = 2 mg/ml) rate of 12.8% was obtained. The antibiotic susceptibility data for all the *S. aureus *isolates are depicted on Table 3. Large proportions of MSSA isolates were still over 85% sensitive to several antimicrobial agents available and readily used in the country.

**Table 3:** Antibiotic susceptibility pattern of 1912 *Staphylococcus aureus *isolates from clinical specimens received at three major regional hospitals in Trinidad & Tobago, 2000–2001 (%)

Antimicrobial	Percent of isolates sensitive	
	
	MSSA isolates, n = 1668 (87.2)	MRSA isolates, n = 244 (12.8)
Penicillin	86.2	0
Ceftriaxone	87.0	0
98.8	98.8	100
Ciprofloxacin	88.0	13.9
Oxacillin	87.5	0
Erythromycin	85.6	0
Gentamicin	87.6	0
Meropenem	91.3	13.9
Tetracycline	85.7	13.9
Trimethoprim/Sulphamethazole	87.0	13.9
Rifampin	100	100
Vancomycin	100	100

n = total number of the isolates; MSSA = methicillin sensitive *S. aureus*; MRSA = methicillin resistant *S. aureus*

All the MRSA isolates were resistant to ceftriaxone, erythromycin and gentamicin, but were 100% sensitive to vancomycin, rifampicin and chloramphenicol. No antibiotic resistance pattern was found to be specific to any particular ward (data not shown)

## Discussion

In this study, the prevalence of methicillin resistant *Staphylococcus aureus *was found to be 12.8% from three major regional hospitals in Trinidad and Tobago. This prevalence is higher than previous rates, 0.7% in 1992–1993; 4.8% in 1995–1996 and 9.8% in 1997–1998 that had been reported in the country [12-15]. This 12.8% prevalence rate found in this study is exactly the same rate found in a pan-European data on methicillin-resistant *Staphylococcus aureus *(MRSA) that was obtained from studies conducted among 43 laboratories from 10 European countries [16]. However, in other countries such as Portugal and Italy, the prevalence was over 50%; England, Greece and France 25%, while the Netherlands and Switzerland had the lowest 2% [17]. Among Western Pacific countries, percentages of MRSA strains ranged from 23.6% in Australia to over 61% in Taiwan and Singapore, and more than 70% in Japan and Hong Kong [18]

This increase of MRSA prevalence found in this study when compared to previous reports in the country may be due to several factors – the length of study period, number of study sites and the sample size. Data from previous research were from only one hospital in the country, were conducted in less than 24 months, and had lower amount of specimens giving isolates of *Staphylococcus aureus*. This study was however not designed to identify risk factors for MRSA prevalence but in a country where prevalence is low, this has been associated with restriction of antibiotic use, strict infection control measures and high ratio of nurses to patients [19]. All these do not exist in these major hospitals in Trinidad & Tobago and could have contributed to the high MRSA prevalence rate during this period of study. This 12.8% prevalence rate in this country is still low compared to rates in the USA or some other countries, and the reason why there may be a progression of MRSA prevalence in the country is not clear and requires further study. In a study conducted elsewhere, a rise in MRSA infections was associated with epidemics in large teaching hospitals which later spread to other areas [20]. There has not been any reported case of MRSA outbreak in these regional hospitals in Trinidad & Tobago.

Prevalence of MRSA in this country as a whole did not vary significantly by gender in this study, and this is in agreement with earlier reports by Saravolatz et al and Charlebois et al [21,22]. These authors have reported that gender was not identified to be a risk factor for the acquisition or colonization of MRSA. In this study however there was a significant difference between the prevalence of methicillin-resistant *Staphylococcus aureus *and methicillin sensitive *Staphylococcus aureus *in both males and females. In a study by Osmon et al, they found no difference in the prevalence of MRSA and MSSA isolates from the cases they studied [23]. No obvious reason has been reported in literature as to the impact of gender in the prevalence of methicillin resistant or sensitive *Staphylococcus aureus *in the community or hospital settings. The probable reason for no difference in gender prevalence of *Staphylococcus aureus *in this study may be population characteristics, with approximately same number of males and females presenting in any particular area with infections. In this our study, at the EWMSC for example, more female patients were seen with MRSA because more females were equally seen at that hospital.

The highest prevalent rates of MRSA isolates were encountered in wound swabs specimens and the surgical wards of the hospitals in this study. These findings are also in agreement with result in Canada by Simor et al, where most of the patients with MRSA were older adults receiving care on medical or surgical units [24]; and also in a study by Müller-Premru and Gubina, where MRSA epidemic occurred in a surgical unit of a hospital [25]. The pattern still has not changed because earlier report by Swanston also noted more MRSA isolates coming from the surgical ward because of high usage of antibiotics in this facility [13]. Methicillin resistant *Staphylococcus aureus *infection on surgical wards is becoming increasingly common especially in critically ill patients who have spent prolonged periods on the intensive care unit [16]. This study was however not designed to identify risk factors for MRSA acquisition, but risk factors that have previously been associated with acquisition of MRSA in hospitals have also included broad-spectrum antimicrobial therapy, admission to an intensive care unit, older age and proximity to other patients with MRSA [26-28].

All MRSA isolates encountered in this study were completely resistant to antibiotics such as penicillin, oxacillin, ceftriaxone, gentamicin and erythromycin. A similar result was noted for penicillin and oxacillin among MRSA strains from some other Latin American countries: thus Brazil (penicillin), Chile (penicillin, oxacillin), Mexico (penicillin, oxacillin) [29]. There is now an increase in resistance pattern for erythromycin and gentamicin because earlier report by Orrett noted that MRSA resistance occurred in less than 70% of the MRSA isolates for these two antimicrobial agents in 1997–1998 [14]. A probably reason for this sustained increase may be due to increase in admission population and perhaps poor infection control measures. Again, this is still an issue that will require further study since this study was not designed to check for reason for increase in antibiotics resistance.

There seems to be a steady rise of MRSA isolates resistance to commonly used antibiotics in the country but fortunately there has been no report of vancomycin resistance. All MRSA isolates in this study were completely sensitive to chloramphenicol, rifampin and vancomycin. A similar result was obtained for vancomycin in previous reports in Trinidad [13,14,30], and MRSA isolates from Argentina, Brazil, Chile, Mexico and Uruguay from 1996 to 1998 were sensitive to vancomycin too [29]. Diekema DJ et al have reported that most MRSA strains are resistant to most other antibiotics, thereby necessitating the use of glycopeptide antibiotics, such as vancomycin [18]. The result of this is treatment failures caused by some strains with decreased susceptibility to vancomycin (vancomycin intermediate *S. aureus*, VISA) that has been reported in Japan and the United States [31,32]. With reports of vancomycin reduced susceptibility and resistance reported elsewhere [33-36], it is imperative that prudent use of vancomycin be restricted to those cases in which it is absolutely necessary, and a continuous surveillance of MRSA activity on vancomycin be carried out regularly in these hospitals in Trinidad. A report from Jamaica indicates that there is no vancomycin resistance in a tertiary hospital there and this is because there is antibiotics (including vancomycin) policy in that institution [37].

The limitation to this study included the lack of knowledge of some risk factors associated with incidence and prevalence of MRSA infections in these hospitals

## Conclusion

There seems to be a progressive increase in MRSA prevalence in the country, however, the rate in the country is low in comparison to the rate in some other countries. Vancomycin is still the drug of choice for treating multidrug resistant MRSA infections. To combat this progressive MRSA prevalence trend in these hospitals, a regular surveillance of hospital associated infection, monitoring of antibiotic susceptibility pattern and formulation of definite antibiotic policy may be helpful. Further use of molecular studies to monitor the epidemiology of MRSA in these hospitals in the country is highly recommended too.

## Competing interests

The author(s) declare that they have no competing interests.

## Authors' contributions

APE coordinated and participated in design of the study and helped also in the drafting of the manuscript. KS conceived the study, participated in its design and coordination and helped draft the manuscript. SWH designed the study, participated in the coordination and helped in the draft of the manuscript. MM participated in the design and coordination of the study. All authors read and approved the final manuscript.
